# Let the female tract choose: finding the sperm to enhance *in vitro* embryo production

**DOI:** 10.1590/1984-3143-AR2026-0061

**Published:** 2026-07-27

**Authors:** Marie Saint-Dizier, Luna Ehrhardt, Alba Perez-Gomez, Joanna Maria Gonçalves Souza-Fabjan, Coline Mahé

**Affiliations:** 1 INRAE, CNRS, University of Tours, PRC, Nouzilly, France; 2 Faculdade de Veterinária, Universidade Federal Fluminense – UFF, Niterói, RJ, Brasil

**Keywords:** oviduct, IVEP, fallopian tube, uterus, microfluidics

## Abstract

*In vitro* embryo production (IVEP) has become an essential tool for livestock breeding, genetic dissemination, and research. Despite its widespread use, overall efficiency remains limited, as only approximately 25% of fertilized oocytes develop into transferable embryos, and pregnancy outcomes are consistently lower than with *in vivo*-derived embryos. While oocyte quality is a major determinant, fertilization success and early embryonic development are also strongly influenced by sperm functional competence, which is only partially captured by conventional selection methods based primarily on motility and morphology. *In vivo*, the female reproductive tract imposes stringent physical and molecular barriers, with fewer than 0.01% of inseminated sperm reaching the fertilization site. This selection involves key processes, including i) passage through the utero-tubal junction, ii) formation and regulation of the oviductal sperm reservoir, and iii) guided sperm navigation toward the oocyte. These mechanisms ensure functional selection of sperm and synchronized meeting with oocytes, features that are largely absent or poorly replicated in conventional *in vitro* fertilization (IVF) systems. Current sperm selection methods include swim-up and density gradient centrifugation. Emerging biomimetic approaches aim to better replicate physiological conditions, notably through microfluidic platforms and oviduct cell–based selection systems. These approaches have shown promising improvements in IVEP, although their routine application remains constrained by limited throughput, standardization, and scalability. This review synthesizes current knowledge on both the *in vivo* mechanisms of sperm selection and emerging biomimetic techniques, particularly in cattle, where improving sperm selection remains a key challenge for IVEP efficiency.

## Introduction

*In vitro* embryo production (IVEP), comprising oocyte recovery, *in vitro* maturation (IVM), fertilization (IVF), and embryo culture, is a major tool for the dissemination of superior female genetics, germplasm conservation, and fundamental and applied research in mammals. In 2024, over two million cattle embryos were produced *in vitro* worldwide, more than sevenfold the number produced *in vivo*, as reported by the International Embryo Transfer Society (Viana, 2025). The global increase in the use of IVEP in farm animals underscores the need to further optimize its outcomes. However, the process remains relatively inefficient, as only about 25% of *in vitro*-fertilized oocytes develop into transferable embryos in cattle (Viana, 2025). In addition, *in vitro* produced embryos result in approximately 20% lower pregnancy rates than those achieved with *in vivo* embryos (Lonergan and Fair, 2014; Ealy et al., 2019; Hansen, 2019). It has been estimated that only 27% of cows receiving IVF embryos produce a live calf (Ealy et al., 2019).

Cleavage rates, often evaluated 2 days post-IVF, are usually high, around 80-90%. However, cleaved embryos include parthenotes (which cleave despite no fertilization) and polyspermic embryos, both of which have compromised development. When IVF zygotes were further examined for the number and origin of pronuclei, the rates of normal monospermic IVF were below 70% in cattle (Elliott et al., 2009). In goats (Bragança et al., 2021) and pigs (Gil et al., 2004; Canovas et al., 2009; Batista et al., 2016), the fertilization rate is usually between 10 and 60% due to a high rate of polyspermy. Oocyte developmental competence is a major biological constraint limiting IVEP efficiency in mammals, yet, fertilization success and subsequent embryo development are also directly influenced by sperm quality (Ward et al., 2001; Alcântara-Neto et al., 2020; Vallet-Buisan et al., 2023). Spermatozoa not only deliver the paternal haploid genome at fertilization but also provide a range of molecular and structural components, such as proteins, regulatory RNAs, centrioles, and epigenetic marks, which can modulate further embryonic development.

*In vivo,* the female reproductive tract imposes several physical and molecular barriers that are absent in conventional IVF systems. Recently, new sperm selection approaches that mimic the sperm journey through the oviduct have shown promising IVF outcomes. The first section of this review will describe the *in vivo* mechanisms governing sperm selection during migration across the female reproductive tract, with a particular focus on post-uterine sperm selection. Mechanisms of sperm selection in the cervix were recently reviewed (Fair et al., 2019; Warr et al., 2023) and will not be addressed here. The second section will focus on emerging sperm selection methods used prior to IVF, especially in cattle: the microfluidic chips designed to exploit sperm behavioral traits in the oviduct (rheotaxis, thigmotaxis, chemotaxis, or thermotaxis) and oviduct-inspired models.

## *In vivo* mechanisms of sperm selection in the female tract

The mechanisms of sperm migration are generally difficult to study *in situ* because the female reproductive tract is inaccessible without invasive procedures. A primary criterion for evaluating sperm migration is the sperm recovery rate, defined as the number of sperm recovered from a specific part of the tract relative to the number initially deposited via insemination. This recovery rate varies significantly depending on the time interval following insemination, the site of deposition, and among females (**Table 1**). While sperm counts can be performed after uterine flushing in big farm animals like cattle and horses, recovering oviductal contents requires surgery or slaughter, which significantly limits the number of studies and the number of individuals per study. Almost all *in vivo* studies of migration in the oviduct provide static post-mortem or post-surgical images and do not accurately reflect the dynamic processes involved. Furthermore, majority of sperm in the oviduct are bound to the luminal epithelial or buried deep within very narrow mucosal folds (Sostaric et al. 2008; Ryu et al., 2024), which underestimates the recovery rate. Thus, the *in vivo* sperm counting data should be interpreted with caution.

**Table 1:** Rates of sperm selection by the female tract after natural mating (NM) or artificial insemination (AI) in cattle.

**AI or NM**	**Semen deposition**	**Time post-AI or NM**	**Number of sperm inseminated**	**Number of sperm (% inseminated) in each part of the female tract**						**Reference**
				**Uterus**	**Utero-tubal junction**	**Lower isthmus**	**Upper isthmus**		**Ampulla**	
NM	Vaginal	2 h			2 - 171	0 - 6	0		0	(Heyman and Levasseur, 1981)
		8 h			3,857 – 4,674	51 - 666	0 - 42		0 - 56	
		18 h			20,000 – 43,686	0 – 2,848	0 - 938		6 - 10	
		48 h			94 – 3,380	279 - 342	0 - 104		12 - 63	
		72 h			179 – 2,947	8 - 232	0 – 1,399		0 - 186	
		19 – 22 h		1,400,000		790				(Mattner, 1968)
		24 – 48 h			200	30-50	<10			(Hunter et al., 1991)
AI	External ostium of cervix	1 h	2,000,000	2,900,000 ± 200,000 (0.15%)	40,000 ± 6,000 (0.002%)	24,000 ± 5,000 (0.001%)				(Dobrowolski and Hafez, 1970)
		8 h	2,000,000	5,300,000 ± 600,000 (0.27%)	150,000 ± 50,000 (0.008%)	200,000 ± 40,000 (0.01%)				
		24 h	2,000,000	2,700,000 ± 100,000 (0.14%)	60,000 ± 2,000(0.003%)	15,000 ± 4,000 (0.0008%)				
AI	Uterine	3-30 min	300,000,000	35,000,000(11.7%)	30,000 (0.01%)					(Suga and Higaki, 1971; Hawk, 1987)
		30-60 min		13,000 (0.004%)	142 (0.00005%)					
		1-2 h		7,000 (0.002%)	21 (0.000007%)					
		2-3 h		2,000 (0.0007%)	194 (0.00007%)					
		3-5 h		300 (0.0001%)	319 (0.0001%)					
		2 h	160,000,000	16,100 – 266,100 (0.01 – 0.17%)	2,600 – 21,700 (0.002 – 0.014%)	0 – 15,600 (0 – 0.01%)		2,600 – 4,500 (0.002 – 0.003%)		(Larsson and Larsson, 1985)
		12 h		0 – 202,800 (0 – 0.13%)	0	1,700 – 21,700 (0.001 – 0.014%)		0 – 20,000 (0 – 0.013%)		

The sperm count ranges listed are the sums of the two uterine horns or the two oviducts.

In cattle, maximal numbers of sperm in the oviducts were observed within 8 to 48 h after mating or artificial insemination (AI; **Table 1**). After AI, the sperm recovery rate was systematically less than 0.01% of inseminated sperm (Dobrowolski and Hafez, 1970; Heyman and Levasseur, 1981). Studies in mice have also reported a decreasing gradient of sperm numbers from the uterine horns to the oviducts, to reach only a few dozen to hundreds in the ampulla, the site of fertilization (La Spina et al., 2016; Nagashima et al., 2019). Further analysis of sperm retrieved in the utero-tubal junction (UTJ) and oviducts of pigs (Garcia-Vazquez et al., 2016) and mice (Kawano et al., 2014; García-Vázquez et al., 2015; La Spina et al., 2016; Muro et al., 2016) indicated that more than 95% were of normal morphology and with an intact acrosome. What mechanisms are in place to ensure that only the fittest spermatozoa proceed toward the oocytes?

Three main biological parameters govern sperm migration in the female tract: the initial quality of the semen in terms of motility, morphology, and resistance to osmotic stress; the mechanical forces and fluid flow generated by the contractions and ciliary beating of the female tract; and cellular and molecular interactions between sperm and female epithelial cells and their secretions. Sperm-female cell interactions occur throughout the female reproductive tract, but their functions vary significantly depending on the location: sperm elimination is the main vocation of the uterus, primarily through contractions and phagocytosis, while sperm survival is handled by the UTJ and oviducts, through the formation of a “functional sperm reservoir”.

## Uterine clearance mechanisms: contractility and innate immune surveillance

Uterine contractions increase during estrus and may also be further stimulated by the presence of sperm (Bourke and Lindsay, 1988; Katila, 2001). A substantial proportion of sperm is eliminated by retrograde uterine contractions within 1 to 2 h post-insemination, a process called semen backflow (Suga and Higaki, 1971; Larsson and Larsson, 1985; Hawk, 1987). Retrograde flows contain normal and motile sperm, but seem to be enriched in abnormal sperm containing cytoplasmic droplets and tail defects, as compared to the ones found at the tip of uterine horns (García-Vázquez et al., 2015). This suggests an initial selective process within the female reproductive tract. The remaining spermatozoa are perceived as foreign invaders in the uterus and trigger a maternal innate immune response, involving macrophages and polymorphonuclear neutrophils (PMNs) (Alghamdi et al., 2009; Tecle et al., 2019; Marey et al., 2020). In cattle, PMNs increase within 2 to 6 h after AI in the uterine lumen and eliminate sperm through direct phagocytosis and the formation of neutrophil extracellular traps (NETs) (Alghamdi et al., 2009; Zambrano et al., 2016; Marey et al., 2023; Rivera-Concha et al., 2023). Incubation of sperm with PMNs triggered NETs formation within 3 h, resulting in a net reduction in progressive motility compared to controls in humans (Zambrano et al., 2016). This interaction is modulated by the presence of the seminal plasma, which tends to enhance the inflammatory response and sperm-PMNs binding (Alghamdi et al., 2009). *In vitro* studies in cattle and humans indicate that PMNs target sperm with a premature capacitation rather than non-capacitated ones (Oren-Benaroya et al., 2007; Rivera-Concha et al., 2023), suggesting a second selective process of uterine clearance.

## The utero-tubal junction: a molecular checkpoint for sperm entry in the oviduct

**Figure 1:**
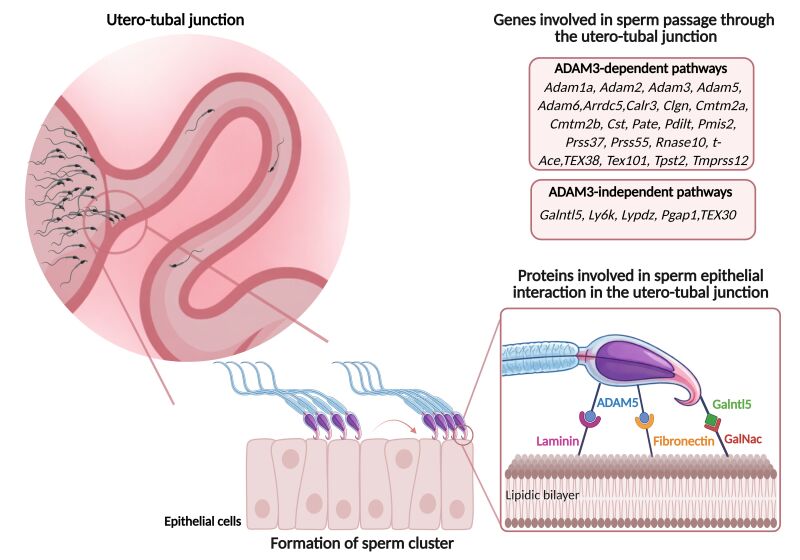
**Mechanisms of sperm selection through the utero-tubal junction (UTJ) in mice.** The UTJ acts as a major anatomical and molecular barrier regulating sperm entry into the oviduct. Its narrow lumen and complex mucosal folds restrict sperm passage, allowing only a small subpopulation to progress. In mice, sperm transit through the UTJ depends on specific behaviors, including the formation of sperm clusters, and involves interactions between sperm surface proteins and epithelial ligands. BioRender has granted L. Ehrhardt permission to use the figures in accordance with BioRender's Terms of Service and Academic License Terms (https://BioRender.com/).

The UTJ represents the first major anatomical barrier encountered by sperm inseminated in the uterus. This region is characterized by a significant constriction in diameter, combined with a maze-like arrangement of mucosal folds that extend into the lumen (Yániz et al., 2000; Suarez, 2008) and mucin-rich viscous secretions (Rickard et al., 2019), which together form a stringent physical barrier to sperm progression. In mice, the entrance of the oviduct is so tight that only a few spermatozoa could pass through a gap between two mucosal folds at a time, and always in the head-forward direction (Ryu et al., 2024). Beyond the physical barrier, sperm must present a molecular passport to be able to cross the UTJ, at least in mice. The sperm passage through the UTJ has been most extensively studied in the mouse, first for a practical reason: the mouse oviduct has a thin wall, making it nearly transparent after tissue clearing techniques are applied. When combined with fluorochrome-labeled sperm, this approach allows precise tracking of sperm progression and behavior, although it typically requires female euthanasia. A second advantage of mice is the availability of genetically modified knockout models. To date, 27 genes have been identified in mice as critical for sperm to reach the oviducts (Fujihara et al., 2018; Fujihara et al., 2019; Xiong et al., 2019; Larasati et al., 2020; Mashiko et al., 2026; Noda et al. 2025; Yuan et al., 2025; Kikuchi et al., 2026) (**Fig. 1**). Male mice lacking any of those genes have severe infertility despite exhibiting normal sperm morphology and motility, underscoring the importance of specific molecular interactions with the female tract. Among these genes, 22 are involved in the expression, maturation, and membrane localization of the Disintegrin and metalloproteinase 3 (ADAM3) (Yamaguchi et al., 2009; Fujihara et al., 2018; Xiong et al., 2019). The protein ADAM3 is also needed for sperm binding to the zona pellucida (Yamaguchi et al., 2006), indicating shared mechanisms between sperm migration into the oviduct and the ability to fertilize oocytes. The remaining genes identified in mice as required to cross the UTJ (*Galntl5, Ly6k*, *Lypd4*, *Pgap1* and *Tex30)* are not linked to ADAM3 localization, indicating ADAM3-independent mechanisms (Fujihara et al., 2018; Noda et al. 2025; Kikuchi et al., 2026).

The interaction mechanisms by which sperm cross the UTJ remain poorly understood. Thanks to live imaging, the particular hook form of the mouse sperm head was recently shown to play a major role in entering the oviduct, by anchoring to the luminal epithelium of the UTJ (Ryu et al., 2024) (**Fig. 1**). Probably helped by their particular head shape, mouse sperm in the UTJ form clusters of dozens of sperm oriented in the same direction, that exhibit synchronized flagellar beating (Qu et al., 2021; Ryu et al., 2024): this cooperation-based behavior may increase their driving force to cross the UTJ. However, improved motility associated with sperm aggregation has been reported only in rodents and some marsupials (Moore and Taggart, 1995; Qu et al., 2021; Ryu et al., 2024), with no clear demonstration in livestock species.

At the molecular level, the sperm-surface protein ADAM5, among proteins needed to cross the UTJ, was shown to interact with laminin I and fibronectin (Mashiko et al., 2026), two proteins primarily present in the extra-cellular matrix but also reported at the surface of the oviduct lumen (Osycka-Salut et al. 2017). In addition, the sperm protein GALNTL5 was shown to interact with the UTJ surface via binding to N-acetylgalactosamine (GalNAc) residues (Noda et al. 2025). Interestingly, while *Adam3* and *Adam5* are pseudogenes in non-rodent mammals, *Galntl5* is widely conserved among eutherian mammals, including humans and cattle. It is thus possible that sperm interactions with surface carbohydrates are conserved mechanisms governing sperm transit into the oviduct. Consistent with this notion, blocking the GalNAc binding sites decreased sperm binding to epithelial cells lining the UTJ in llamas (Apichela et al., 2010).

This molecular passport may prevent unfit spermatozoa, as well as those undergoing a premature capacitation, from reaching the oviduct. In mice, after mating, sperm in the isthmus exhibit both a higher proportion of intact acrosomes (Kawano et al., 2014; Muro et al., 2016) and lower tyrosine phosphorylation levels (a capacitation marker) (Ded et al., 2020) compared to those in the uterus. The selection by the UTJ may extend to chromatin quality: spermatozoa recovered from the oviduct exhibited lower DNA fragmentation than those in the uterus (Hourcade et al., 2010). Together, these findings highlight the role of the UTJ as a multilevel gatekeeper against sperm with structural, functional, and DNA abnormalities.

## Formation of an oviductal sperm reservoir: survival of the fittest

**Figure 2:**
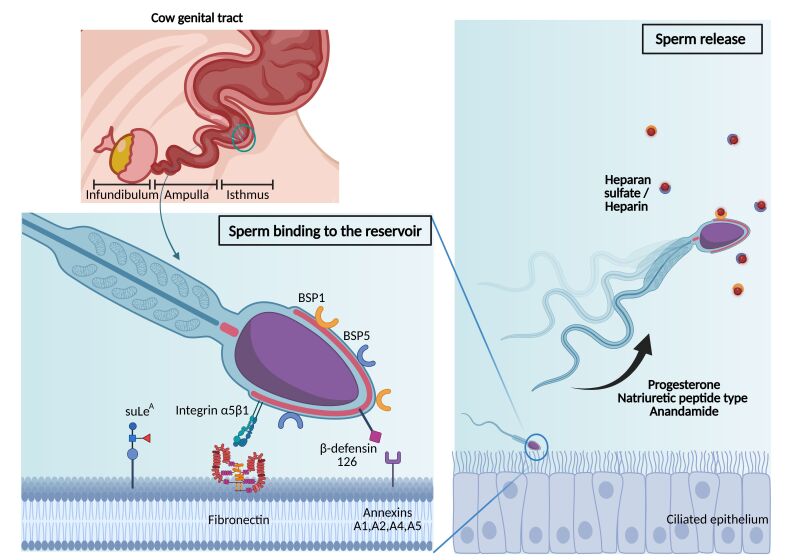
**Mechanisms of sperm selection through the formation of a reservoir and release toward the oocyte in the cow oviduct.** After crossing the utero-tubal junction, a subpopulation of spermatozoa binds to the ciliated epithelium of the isthmus, forming a functional sperm reservoir. Binding is mediated by interactions between sperm proteins and oviduct epithelial components, including annexins, fibronectin, and glycan motifs. Sperm release is trigggered by peri-ovulatory signals that promote detachment through hyperactive motility and progression toward the fertilization site. BioRender has granted L. Ehrhardt permission to use the figures in accordance with BioRender's Terms of Service and Academic License Terms (https://BioRender.com/).

After crossing the UTJ, sperm reaches the isthmus, the first part of the oviduct, where a subpopulation binds stably by their head to the luminal cilia, forming a “functional sperm reservoir” (Suarez, 2008; Miller, 2018, 2026) (**Fig. 2**). Several studies reported that only sperm with active flagellar beating and presenting an intact acrosome with no morphological abnormality are able of binding to the oviduct epithelium (Sostaric et al. 2008; Camara Pirez et al., 2021; Gimeno et al., 2021; Mahé et al., 2025b). In heifers mated at the onset of estrus, sperm may be retained for 18 to 24 hours in the isthmus (Wilmut and Hunter, 1984; Hawk, 1987). Similarly, in gilts mated early in estrus, sperm may survive for 36 hours or more in the caudal isthmus (Hunter, 1984). Beyond extending sperm lifespan, the sperm reservoir is thought to synchronize gamete encounters and reduce the risk of polyspermy by facilitating the gradual release of sperm towards the ampulla (Miller, 2018; Saint-Dizier et al., 2025). The mechanisms underlying the formation of the reservoir are not yet fully understood. On the female side, sperm interaction relies on specific apical proteins, including for the bovine species the non-glycosylated annexins A1, A2, A4, and A5 (also called Annexin V) (Ignotz et al., 2007), and glycan motifs, including the 3'-O-sulfated form of Lewis A trisaccharide (Kadirvel et al., 2012; Machado et al., 2014; Dutta et al., 2019) (**Fig. 2**). High-throughput proteomics data have led to recent advances in this field. The analysis of the bull sperm-interacting proteome in the oviduct, coupled with the analysis of the apical cilia of the bovine oviduct epithelium, allowed us to predict 19 new candidates involved in reservoir formation (Mahe et al., 2023a, b). On the sperm side, ligands to cilia may include a variety of proteins, including the integrin α5β1 (Osycka-Salut et al. 2017) originated from the testis, the epididymal β-defensin 126 (DEFB126) (Tollner et al., 2008; Lyons et al., 2018), and seminal proteins coating the sperm surface at ejaculation, called the binder of sperm proteins (BSP 1,3 and 5) in cattle (Gwathmey et al., 2003, 2006) (**Fig. 2**).

## Sperm release from the reservoir: responsiveness to female signals and capacitation required

The timed release of spermatozoa from the reservoir toward the ampulla, where cumulus-oocyte complexes (COCs) advance following ovulation, is essential for fertilization (**Fig. 2**). In cattle, there is evidence that sperm release from oviduct epithelial cells (OECs) is mediated by progesterone through membrane receptors and CatSper-dependent calcium influx (Lamy et al. 2017; Romero-Aguirregomezcorta et al., 2019; Ramal-Sanchez et al., 2020). Sulfated glycosaminoglycans, like heparan sulfate and heparin, are probably involved in sperm release through the detachment of BSPs from the sperm head (Gualtieri et al., 2010; Ramal-Sanchez et al., 2020; Mahe et al., 2023b). Levels of both progesterone and sulfated glycosaminoglycans increase in the oviduct fluid at estrus (Bergqvist and Rodriguez-Martinez, 2006; Lamy et al. 2016). Additional factors such as anandamide (Gervasi et al., 2016; Kumar et al., 2017) and the natriuretic peptide type C (NPCC) (Wang et al., 2022; Wu et al., 2023) may also play roles in sperm release from the reservoir at ovulation (**Fig. 2**).

The oviduct is a milieu inducing sperm capacitation (Parrish et al., 1989), a series of metabolic and physical changes leading to acrosome reaction (for review, see (Malverdi et al., 2026). *In vivo* imaging of mouse sperm progressing through the oviduct showed an increase in the proportion of sperm with a reacted acrosome, from around 2% in the lower isthmus to 95% in the ampulla (Kawano et al., 2014; La Spina et al., 2016; Muro et al., 2016; Ded et al., 2020). In cattle, sperm released from OECs by the action of progesterone or heparin still present an intact acrosome (Bosch et al., 2001; Lamy et al., 2017), indicating that sperm release is not triggered by an advanced capacitation status with the loss of the acrosomal membrane. However, it is likely that the release from the reservoir is linked to the initiation of capacitation. The comparison between bound-released and control unbound bull sperm evidenced among the released population a higher progressive motility, elevated levels of intracellular calcium (Osycka-Salut et al. 2017), and higher membrane fluidity (Ramal-Sanchez et al., 2020), all signs of sperm ongoing capacitation. The sequence of events at the moment of release is however not entirely clear: progressive capacitation of some sperm may trigger their release, or female signals trigger first the release, which, in turn, causes capacitation.

## Sperm navigation in the oviduct: integration of physical and chemical female signals

Several facts prompted the search for mechanisms to guide sperm toward the fertilization site. First, the number of sperm reaching the isthmic reservoir is, as seen above, very small. Second, the distance between the isthmus and the ampulla is relatively long, around 15 to 20 cm in cattle. The overall length of bull sperm being on average 70 µm, this path is 2,000 to 3,000 times higher. Third, the oviduct lumen is a labyrinth filled with highly complex mucosa folds (Yániz et al., 2000), at the bottom of which one egg cell (in humans and cows) may be hiding. Last, matured oocytes have a limited period of fertility, estimated around 6 h in cows, which means that sperm guiding should be efficient and relatively rapid, especially if insemination is performed close to ovulation time.

**Figure 3:**
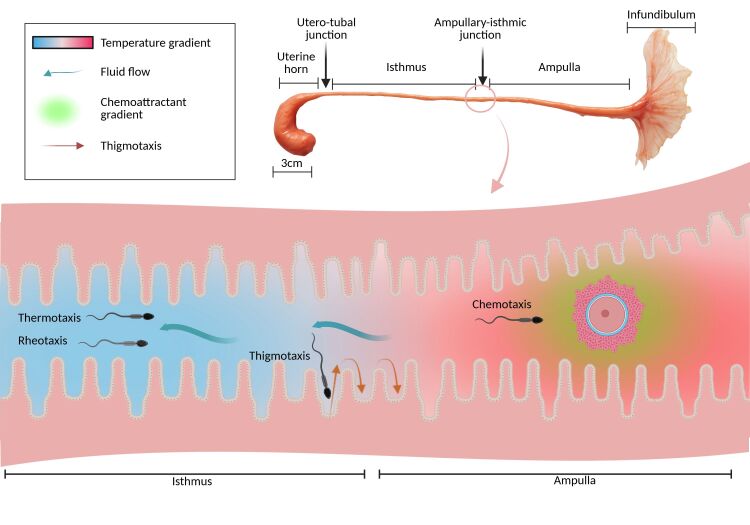
**Proposed model of sperm guidance in the cow oviduct.** Spermatozoa navigate through the oviduct by integrating multiple physical and chemical cues, including rheotaxis (orientation against fluid flow), thigmotaxis (wall-following behavior), thermotaxis (migration along temperature gradients), and chemotaxis (response to chemoattractants). BioRender has granted L. Ehrhardt permission to use the figures in accordance with BioRender's Terms of Service and Academic License Terms (https://BioRender.com/).

There is evidence that sperm are able to orient and swim in response to some physical and chemical external stimuli: these particular behaviors include rheotaxis, thigmotaxis, thermotaxis and chemotaxis (**Fig. 3**), and have been proposed as key players of sperm guidance in the oviduct (Li and Winuthayanon, 2017; Eisenbach, 2025). A positive rheotaxis is the ability of a cell or an organism to orient and swim against a fluid flow. This capacity was reported in spermatozoa of several mammals, including mice, humans (Miki and Clapham, 2013), and cattle (El-Sherry et al., 2014; Tung et al., 2014; Johnson et al., 2017; Romero-Aguirregomezcorta et al., 2021; Yazdan Parast et al., 2023). By the combined action of oviductal muscular contractions and ciliary beating of the oviduct epithelium, it is likely that sperm encounter a fluid flow directed mainly from the ovary toward the uterus (Miki and Clapham, 2013; Hino and Yanagimachi, 2019), introducing an additional selective process of migration by rheotaxis. This flow was measured at 18 µm/s in the mouse ampulla (Miki and Clapham, 2013).

Thigmotaxis is defined as a behavioral trait characterized by the avoidance of open areas, preferring to remain near walls like rodents in open field tests. Human sperm thigmotaxis was evidenced 15 years ago using microchannels of various geometries (Denissenko et al., 2012). Using this device, it was demonstrated that sperm preferentially swims along the channel walls instead of in their central part. When encountering an abrupt bend, sperm detach from the corner and continue swimming forward until they reach the opposing wall. This behavior is thought to allow sperm to swim more efficiently and rapidly through the microgrooves of the cervix, UTJ and oviduct lumen. Another study evidenced that normal sperm could swim much faster than those with abnormal morphology in microfluidic systems with micropillars (Chinnasamy et al., 2018), opening possibilities for sperm sorting based on thigmotaxis. However, it is important to note that data on sperm thigmotaxis in farm animals remain limited.

Capacitated sperm can be also guided by thermotaxis and chemotaxis toward the COC (**Fig. 3**) (Eisenbach, 1999; Xiao et al., 2022). Spermatozoa are extremely sensitive to temperature variations. Human sperm can sense and respond to temperature differences as low as 0.01 °C/mm, and their thermotactic responsiveness has been observed over a wide range of temperatures (29 to 41°C) (Eisenbach, 2025). Thermotaxis can be considered as a long-range guiding mechanism that guides sperm from the reservoir to the warmer ampulla. The ampulla at estrus was found to be 0.7 °C warmer than the isthmus in sows (Hunter and Nichol, 1986) and 2 °C warmer in rabbits (Bahat et al., 2003). Using a two-chamber system (35-36 °C and 39 °C) connected by a capillary, sperm thermotaxis was observed in cattle (Ruiz-Díaz et al., 2023), mice, and humans (Pérez-Cerezales et al., 2018), although the proportion of responsive sperm was limited (0.5 to 4%). Sperm thermotaxis relies on a family of membrane G-protein-coupled proteins that can sense either light or temperature, depending on the cell type: opsin-2, -3, -4, and -5 are present on human and mouse sperm, but also in their eyes (Eisenbach, 2025). Opsin-2 and Opsin-4 were shown to be crucial for sperm to perform thermotaxis, each mediating different signaling pathways (Roy et al., 2020).

Chemotaxis has been proposed as a short-range guiding mechanism near the oocyte (Eisenbach, 1999). *In vitro*, human sperm swim toward picomolar gradients of progesterone (Oren-Benaroya et al., 2008). Progesterone, secreted by the cumulus cells surrounding the oocyte, has been proposed as the main sperm chemoattractant (Li and Winuthayanon, 2017; Eisenbach, 2025). The existence of a picomolar gradient of progesterone is unlikely in the oviduct of cows as intra-oviductal concentrations of progesterone are at least a thousand times higher, from 20 to 200 nM, in the peri-ovulatory period (Lamy et al. 2016). However, using a 2-chamber system, a chemotactic response to progesterone from 1 to 100 pM has been reported in bull (Dominguez et al., 2018), mice (Guidobaldi et al., 2017), and human (Oren-Benaroya et al., 2008) sperm, with proportions of responsive sperm ranging from 10 to 35%. Finally, chemotaxis toward progesterone, as well as rheotaxis and thermotaxis, rely on sperm motility and are largely mediated by CatSper-dependent calcium influx (Miki and Clapham, 2013; Eisenbach, 2025).

## Mimicking the female tract to improve IVEP outcomes

Conventional methods of sperm sorting before IVEP include density gradient centrifugation (DGC) and swim-up. In humans, other sperm selection methods have been tested for ICSI or IVF, including hyaluronic binding assays and Annexin V magnetic-activated cell sorting (MACS), with inconsistent results (for reviews, see (Huszar et al., 2007; Teijeiro et al., 2017)).

The simple and cost-effective swim-up technique remains one of the most widely used method of sperm preparation in IVF laboratories and relies on sperm motility. In practice, a semen sample is placed at the bottom of a tube filled with an appropriate volume of medium. Then, within around 30 minutes, the most motile sperm are recovered in the overlayer. On the other hand, DGC separate sperm based on morphology. The mechanism is essentially a physical filtration process based on cell density. Morphologically normal sperm have a slightly higher density than abnormal ones: they pass through the gradient and form a pellet, while lower-quality sperm and cell debris remain in upper fractions or at interfaces. Sperm processed by DGC are generally more motile due to the selection of an already highly motile and structurally intact subpopulation. The sperm pellet recovered after DGC is usually washed and centrifuged before used for IVF. The rates of recovery and quality parameters of bull sperm sorted using swim-up versus DGC are presented in **Table 2**. Overall, DGC allows to collect more cells and with higher progressive motility than swim-up. However, there are discrepancies among studies concerning sperm membrane and DNA integrity, and cleavage and blastocyst rates after IVF using sperm sorted by both methods.

**Table 2:** Recovery rates, quality and IVEP outcomes of semen after density-gradient centrifugation (DGC) or swim-up in cattle.

**Recovery rate (quantity or concentration recovered)**		**Progressive motility**		**Membrane integrity**		**DNA integrity**		**Cleavage rate**		**Blastocyst rate**		**Reference**
**DGC**	**Swim-up**	**DGC**	**Swim-up**	**DGC**	**Swim-up**	**DGC**	**Swim-up**	**DGC**	**Swim-up**	**DGC**	**Swim-up**	
18.3 ± 3.9%* (4.8 ± 1.0 M)	6.5 ± 0.5% (1.7 ± 0.1 M)	57.1 ± 12.2%*	43.4 ± 10.3%	74.67 ± 5.37 *	67.0 ± 8.1	83%	88%*	83.8 ± 5.1%*	75.2 ± 7.6%	28.1 ± 5.4%*	21.9 ± 4.3%	(Vega‐Hidalgo et al., 2022)
39.8 ± 4.2%* (15.9±1.4 M)	8.5 ± 1.0% (6.8 ± 0.8 M)							42 ± 1%	60 ± 1%*	35 ± 4%	29 ± 1%	(Parrish et al., 1995)
79-84%* (8.5 – 9.5 M)	48 -49% (3.1 – 4.0 M)	49 - 55%	77 – 80%*	86 – 90%	95 – 96%*	91%	93 - 94%	62 -67%	73 - 76%*	8 - 12%	12 - 14%	(Ben et al., 2013) a
		70.0 ± 3.5%*	53.8 ± 3.2%	72.7 ± 2.8%*	51.0 ± 2.2%			77.3 ± 2.0%	72.6 ± 4.0%	**31.8 ± 0.7%**b^,^*	21.9 ± 2.5%^b^	(Samardzija et al., 2006)
				88.2 ± 4.8	69.4 ± 7.5							(Somfai et al., 2002)
		50.0 ± 2.7% (B)* 60.8 ± 1.5% (P)*	49.5 ± 1.0%	89.8 ± 0.9% (P)* 83.3% ± 1.3% (B)	75.0 ± 1.6%	98 - 99%	**99%**					(Arias et al., 2017)
								71%	73%	28%	33%	(Lucio et al., 2012)

a This study was conducted in the yak species; ^b^Include rates of morulas and blastocysts. M, million.

For each parameter, data are presented as means ± SEM or range of values. Asterisks indicate significant differences between DGC and swim-up, with bold letters for the better results. M, million; P, Percoll; B, BoviPure.

## Microfluidic platforms for active biomimetic sperm selection

**Figure 4:**
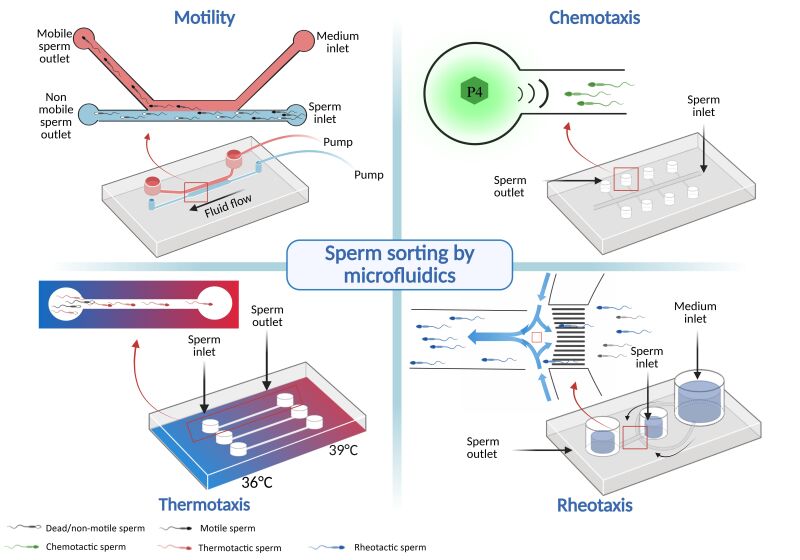
**Sperm sorting microfluidic chips based on sperm natural behaviors in the female tract.** Microfluidic devices enable sperm sorting by exploiting behavioral responses observed in the female tract, including motility (Phiphattanaphiphop et al., 2020), chemotaxis (Li et al., 2016), thermotaxis (Ruiz-Díaz et al., 2023), and rheotaxis (Nagata et al., 2018). BioRender has granted L. Ehrhardt permission to use the figures in accordance with BioRender's Terms of Service and Academic License Terms (https://BioRender.com/)

Microfluidics has emerged as a promising strategy to improve conventional sperm selection methods. Microfluidic devices, also called microfluidic chips, are defined as miniaturized systems that allow fluid control at the micrometric scale, typically in channels with dimensions ranging from tens to hundreds of micrometers. Microfluidic devices are very versatile, allowing cell selection based on a wide range of criteria (Huang et al., 2023). In the last decade, numerous chips incorporating sperm guidance mechanisms through the oviduct have been developed, providing innovative approaches for achieving more efficient and physiologically relevant selection processes (**Fig. 4**). There are also devices for passive sperm selection based on sperm physical characteristics, such as shape, size, or surface charge (Huang et al., 2023). Although passive sperm selection is beyond the scope of this review, it should be mentioned that a microfluidic dielectrophoresis (DEP) chip has been successfully used to enrich bovine X-sorted bull sperm (Wongtawan et al., 2020).

In general terms, microfluidic devices with active sorting consist in an inlet chamber, where the semen sample is deposited, connected by channels to an outlet chamber, where the spermatozoa that moved in response to various stimuli can be recovered (Huang et al., 2023) (**Fig. 4**). The majority of currently marketed microfluidic devices has been developed for use in human ARTs and tested only with human sperm. Their effectiveness has been evaluated mostly in terms of motility, DNA integrity and morphology of sorted sperm (Pensabene et al., 2026). One actual important limitation of most microfluidic chips is their absence evaluation in terms of blastocyst yield, pregnancy and birth rates. Fortunately, since sperm taxis properties are largely shared among mammals (Eisenbach and Giojalas, 2006), microfluidics-based sperm selection strategies can be applied to other mammalian species.

The microfluidic devices already tested in cattle are listed in **Table 3** and primarily exploit the motility and rheotactic capacity of bull spermatozoa. The efficiency of the microfluidic devices is mainly evaluated by sperm concentration and motility after sorting. However, several studies have advanced chip validation by exploring additional parameters beyond these standard metrics. In cattle, the effectiveness of the microfluidic Fertile Bovine^®^ chip (Koek Biotechnology) in selecting motile sperm was evaluated by IVF outcomes: higher cleavage (86% vs. 76%) and blastocyst (44% vs. 33%) rates were reported using sorted sperm compared to semen washed by centrifugation (Alkan et al., 2023). In addition, the blastocysts obtained with sorted sperm contained higher numbers of cells in the inner cell mass and trophectoderm, and displayed lower cell apoptosis.

**Table 3:** Microfluidic devices tested for sperm selection and main outcomes in cattle.

**Sperm selection strategy**	**Brief description of the device**	**Fluid flow**	**Main outcomes**	**Reference**
**Motility**	- K-shaped device	Actively-driven by two syringe pumps	- Processing volume and time not shown	(Phiphattanaphiphop et al., 2020)
	- The sample and medium are introduced using syringe pumps into two separate inlets located at one end of the device. Motile sperm can escape the flow and swim towards the outlet, while immotile sperm are carried by the flow into a waste chamber (**Fig. 4**)			
			- 95% motile sperm in the sample processed	
	- Device FERTILE Bovine® (Koek Biotechnology)	No fluid flow	- 2 straws processed in 20 min	(Alkan et al., 2023)
			- Sperm recovered at >1 M /mL	
	- Dual chambered device: first chamber contains a sample inlet and fluid channels separated from the second collection chamber by a microporous membrane.			
			- Tested for IVF: 85% cleavage and 44% blastocyst rates	
			- Better IVF outcomes compared to centrifuged semen	
**Rheotaxis**	- Diffuser-type microfluidic sperm sorter (DMSS)	Passively-driven by hydrostatic pressure	- 0.5 mL bull semen straw (20 M sperm/mL) processed in 30 min	(Nagata et al., 2018)
	- 3 communicating chambers (medium inlet, sperm inlet and outlet)		- 1 mL of sorted semen was collected: ≤ 1M motile sperm/mL	
	- The rheotaxis area is composed by 14 microchannels, that only rheotactic spermatozoa can pass through (**Fig. 4**)		- 95% improvement in DNA integrity (%DFI 0.4 vs. 7.1, in sorted semen and raw sample, respectively)	
			-Semen sorted valid for IVF/AI	
			-Tested for AI: similar pregnancy rates using sorted semen or standard dose (DMSS-sorted insemination dose represent only 5% of the unsorted dose)	
			- Calf born following AI with microfluidic-sorted sperm	
	Sample inlet connected to a waste chamber via a fluid channel with 7 corral-shaped structures inside that trap spermatozoa capable of swimming against the fluid flow	Actively-driven by two syringe pumps	- Different processing times evaluated: 1 s to 45 min	(Zaferani et al., 2018)
			- ∼ 10% of the motile sperm in the raw semen were recovered	
			- 100% progressive motile sperm in the sorted semen	
			- Applicable to IVF	
	Device with a network of 3 rows of 42 parallel triangular structures that disturb the laminar flow and create local shear rate gradients for rheotaxis selection	Actively-driven by a syringe pump	- 100 µL of sample processed in 20-35 min	(Yaghoobi et al., 2024)
			-Sperm recovered at a concentration of ∼ 20 M sperm/mL, >60% motile sperm, <10% DFI	
			- Tested for IVF: higher blastocyst rates using the device (37%) compared to centrifugation (30%)	
	- Device combining radial flow and contracted pathways to generate rheotaxis areas.	Actively-driven by a syringe pump	- 150 µL of sample processed in 12-22.5 min	(Karimi et al., 2026)
			- 11-27% of the motile sperm in the raw semen are recovered	
	-Rheotactic sperm swim toward the flow origin and can be recovered in a central outlet.			
			- 3-8 M motile sperm/mL in the outlet	
			- 99% viable, 93% motile sperm in the sample processed	
**Chemotaxis**	- Central rectangular inlet with six perpendicular microchannels (three on each long side), each terminating in an outlet containing progesterone or COCs (**Fig. 4**)	No fluid flow	- 300 µL of sample at 100 M/mL sperm processed in 30 min	(Li et al., 2016)
			- Higher mitochondrial activity and acrosome intactness in sperm selected by the device compared to swim-up	
			- IVF can be performed directly on the chip, leading to higher penetration, monospermy and blastocyst rates, compared to conventional IVF	
**Thigmotaxis**	- Radial array of 500 parallel microchannels	No fluid flow	- 1 mL of raw semen processed in 20 min	(Nosrati et al., 2014)
	- Spermatozoa swim across high viscosity fluid through narrow microchannels to a central collection chamber		- 100 µL recovered at a concentration of 1 M sperm/mL	
			- Sperm viability ranging from 80 to 100%	
**Thermotaxis**	- Thermotaxis device already tested in mice and humans (Pérez-Cerezales et al., 2018)	No fluid flow	- Processing time 1 h	(Ruiz-Díaz et al., 2023)
			- 3.6% of the sperm loaded showed thermotactic behavior	
	- Device presenting 3 separation units consisting of two chambers at different temperatures (36 to 39 °C) connected by a microchannel (**Fig. 4**)			
			- Selected sperm showed lower SDF level compared to non-migrated sperm	
			- Higher cleavage and blastocyst rates following ICSI with migrated sperm compared to non-migrated sperm	

References are classified according to the sperm sorting strategy (1^st^ column), and then in chronological order. All references used frozen-thawed semen. AI, artificial insemination; DFI, DNA fragmentation index; HDS, High DNA stability; ICSI, intra-cytoplasmic sperm injection; M, million; SDF, Sperm DNA fragmentation

In addition, in cattle, a rheotaxis-based microfluidic device was developed that allowed for subsequent AI (Nagata et al., 2018). Pregnancy rates using sorted frozen-thawed semen were comparable to those achieved with a conventional dose (37.1% vs. 39.7%; n= 35-68 inseminations) and resulted in healthy live births, despite the sorted semen represented only 5% of the standard dose (≤1 vs. 20 million sperm/insemination). Surprisingly, 79% of sorted sperm in fertile doses displayed a sinuous trajectory pattern, in contrast to the conventional idea that the fertilizing sperm are more linear (Nagata et al., 2018). More recently, another rheotaxis-based device testing a range of flow rates reported an increased sperm speed with less DNA fragmentation as the flow rate increased (Yaghoobi et al., 2024). The use of the faster sperm sorted thanks to the device resulted in 24% more blastocysts than using the control sperm just washed by centrifugation (Yaghoobi et al., 2024).

Li et al. (2016) engineered a device for chemotaxis able to select sperm with higher mitochondrial activity and acrosome intactness compared to conventional swim-up. Their device enabled the introduction of COCs, so that IVF could be directly performed on the chip. They reported higher penetration and monospermy rates, which led to higher blastocyst rates (37% vs. 25%), using the device compared to standard IVF (Li et al., 2016). Furthermore, after introduction of COCs directly into the device to perform IVF, higher penetration and monospermy rates, leading to higher blastocyst rates (37% vs. 25%) were obtained compared to standard IVF (Li et al., 2016).

Sperm sorting based on thermotaxis, using a 36-to-39 °C gradient, was also reported, but the sperm recovery rate (3.5%) was too low to perform IVF, being sufficient only for ICSI (Ruiz-Díaz et al., 2023). Although in limited numbers, the sperm selected by thermotaxis had lower DNA fragmentation and higher competence for embryo development compared with non-migrated sperm (32% vs. 8% blastocysts) (Ruiz-Díaz et al., 2023).

Thus, the results reported to date collectively indicate that bull sperm sorting using biomimetic taxis consistently improves fertilization rates and embryo development. However, some microfluidic platforms do not provide sufficient throughput in terms of sperm recovery for routine use in IVEP, or remain technically challenging to implement, particularly those with an external pump for flow control. Expected advancements in microfluidic technologies make it possible to consider IVF-on-a-chip platforms, that consist in a single device that integrates one or several sperm selection mechanisms with addition of IVM oocytes to closer mimic *in vivo* fertilization (Weng, 2019).

While currently evaluated only with human sperm, other microfluidic devices stand out for their high efficiency and operational simplicity, with potential application for IVF or AI in livestock. In this regard, three devices used a flow passively driven by hydrostatic pressure to select sperm by rheotaxis. The first one, a diffuser-type microfluidic sperm sorter, can process approximately 200,000 spermatozoa per minute and samples up to 200 million sperm per mL (Wu et al., 2017). Second, a device with two chambers (top and bottom) separated by a circular polycarbonate filter, enabled the recovery of 30% motile spermatozoa at a concentration 10-15 million per mL (Ataei et al., 2021). More recently, a microfluidic device featuring four selection areas connected to a central collection chamber allowed the recovery of spermatozoa at a concentration of 2 million per mL, of which 88% exhibited progressive motility (Heidarnejad et al., 2025). The implementation and efficacy of these chips for IVEP and AI remain to be tested in cattle.

### Oviduct epithelial and ligand-mediated sperm selection

Another way to improve IVEP relies on the capacity of some sperm to bind to the oviduct sperm reservoir. As stated earlier, only motile sperm with an intact acrosome and absence of morphological abnormalities are capable of binding to OECs (Sostaric et al. 2008; Camara Pirez et al., 2021; Gimeno et al., 2021; Mahé et al., 2025b). *In vitro* models mimicking this interaction — including explants, epithelial monolayers, spheroids and isolated oviduct glycan ligands — consistently demonstrate that sperm binding competence correlates with membrane integrity (Khalil et al., 2006; Leemans et al., 2014; Saraf et al., 2019; Schmaltz et al., 2024; Mahé et al., 2025a), mitochondrial functionality (Nag et al., 2021), and DNA integrity (Ellington et al., 1999; Nag et al., 2021) (**Table 4**). This suggests that adhesion is not merely a retention mechanism for sperm randomly captured by OEC cilia, but a biomarker of sperm quality. Additional studies in cattle reported higher cleavage rates following IVF using sperm sorted by pre-binding to OECs compared to control sperm lacking interaction (Gualtieri and Talevi, 2003; Lamy et al., 2017; El-Sokary et al., 2022; Marco et al., 2025). Notably, the release of bound sperm was either induced by heparin (Gualtieri and Talevi, 2003), a mix of heparin and progesterone (Lamy et al., 2017; El-Sokary et al., 2022), or a mix of heparin and COCs (Marco et al., 2025), indicating that peri-ovulatory signaling originating from the pre-ovulatory follicle and/or COCs may synchronize sperm capacitation with oocyte availability and enhance their developmental competence.

**Table 4:** Oviduct epithelial cell models used for sperm selection and main outcomes in mammals.

***In vitro* model used**	**Species**	**Origin of female cells**	**Sperm preparation**	**Main outcomes**	**Reference**
**Oviduct explants or aggregates**	Cattle	Pubertal cows at post-ovulatory stage – whole oviduct	Frozen-thawed Percoll-gradient washed semen	A mix of bound and unbound sperm displayed higher ZP binding capacity and produced more fertilized COCs than unbound free sperm	(Kon et al., 2009)
	Cattle	Pubertal water buffalos at non-luteal stage – whole oviduct	Frozen–thawed semen	Sperm-binding index is positively correlated with % sperm with intact membrane and with conception rates after AI	(Saraf et al., 2019)
	Cattle	Pubertal cows at non-luteal stage of cycle	Frozen-thawed semen washed by centrifugation	Binding capacity to explants was associated with low sperm DNA fragmentation, intact membrane and high mitochondrial membrane potential	(Nag et al., 2021)
	Cattle	Pubertal cows – isthmic part of the oviduct	Frozen-thawed semen pre-selected by swim-up	Sperm pre-bound to explants produced higher cleavage rates after IVF, compared with control sperm with no cells	(El-Sokary et al., 2022)
	Horse	Pubertal mares at pre-ovulatory stage of cycle	Fresh Percoll-gradient washed semen	Sperm bound to explants had higher % membrane-intact sperm than unbound sperm	(Leemans et al., 2014)
**Oviduct epithelial spheroids**	Cattle	Pubertal cows – isthmic part of the oviduct	Frozen-thawed Percoll-gradient washed semen	Sperm pre-bound to spheroids produced higher cleavage rates after IVF, compared with control sperm with no spheroids	(Marco et al., 2025)
**OEC monolayers**	Cattle	Pubertal cows – whole oviduct	Frozen-thawed Percoll-gradient washed semen	Sperm bound to OECs and then released by heparin displayed higher binding capacity to ZP and cleavage rates after IVF, compared with unbound and control sperm	(Gualtieri and Talevi, 2003)
**OEC monolayers**	Cattle	Pubertal cows at peri-ovulatory stage – whole oviduct	Frozen-thawed Percoll-gradient washed semen	Sperm bound to OECs then released by progesterone produced higher cleavage and blastocyst rates after IVF, compared with control sperm without cells	(Lamy et al., 2017)
	Pig	Cycling gilts - whole oviduct	Fresh Percoll-gradient washed semen	Pre-incubation of sperm with OECs increased the rate of zygotes with 2 pronuclei and reduced polyspermy, compared with controls without cells	(Bureau et al., 2000)
		Cycling gilts - whole oviduct	Fresh Percoll-gradient washed semen	Sperm pre-bound to OECs produced higher penetration rates and nuclear decondensation after IVF than unbound sperm	(Lopez-Ubeda et al., 2017)
	Horse	Mares of unknown status – whole oviduct	Frozen-thawed semen washed by centrifugation	Sperm bound to OECs were motile and presented an intact acrosome	(Gimeno et al., 2021)
	Human	Women at follicular phase of cycle	Fresh semen washed by centrifugation	Sperm bound to OECs had fewer abnormalities in chromatin structure than unbound sperm	(Ellington et al., 1999)
**Oviduct membrane glycan motif**	Pig	Sulfated Lewis X trisaccharide (suLeX)	Fresh semen washed by centrifugation	Sperm co-incubated with soluble suLeX had reduced mitochondrial electron transport chain and intracellular ROS compared with controls	(Hughes et al., 2023)
		Sulfated Lewis X trisaccharide (suLeX) coupled to a glass surface	Fresh Percoll-gradient washed semen	Sperm bound to suLeX then released by COCs produced higher rates of monospermic zygotes, compared with control sperm with no pre-binding	(Soto-Heras et al., 2025)
**OEC membrane proteins**	Human	OEC membrane protein preparation from an immortalized oviduct cell line	Fresh Percoll-gradient washed semen	Sperm treated with OEC membrane proteins had reduced ROS levels and increased superoxide dismutase (SOD) and glutathion peroxidase (GPX) activities compared with controls	(Huang et al., 2013)

References are classified according to the sperm sorting strategy (1^st^ column), species, and then in chronological order. COCs, cumulus-oocyte complexes; IVF, *in vitro* fertilization; OEC, oviduct epithelial cell; ROS, reactive oxygen species; ZP, zona pellucida.

In pigs, pre-binding to OECs increased oocyte penetration rates and the proportion of monospermic zygotes (Bureau et al., 2000; Lopez-Ubeda et al., 2017). This is particularly relevant for porcine IVEP, where polyspermy remains a major limitation. Beyond cellular systems, simpler models using defined oviductal ligands provide mechanistic insights and may reproduce the beneficial effects on sperm lifespan and IVF outcomes. Incubation of boar semen with sulfated Lewis X trisaccharide (suLeX) under capacitating conditions reduced mitochondrial electron transport activity and reactive oxygen species (ROS) production compared with controls (Hughes et al., 2023). Likewise, membrane protein preparations from human OEC lines decreased ROS and enhanced antioxidant enzyme activity in human sperm (Huang et al., 2013). When immobilized on glass, suLeX selectively bound boar sperm that, after release, generated higher rates of monospermic zygotes compared to conventional IVF (Soto-Heras et al., 2025). These data suggest that interaction with oviduct ligands modulates sperm redox homeostasis and capacitation dynamics, potentially restricting premature acrosome reaction, thereby extending lifespan and preserving fertilization competence.

Despite the robustness of these observations, several limitations merit consideration. Interpretation may be limited by the fact that, in some IVF setups, oocytes are incubated together with spermatozoa and OECs, thereby being exposed to OEC-derived factors (Bureau et al., 2000; Lopez-Ubeda et al., 2017; El-Sokary et al., 2022). This confounds the attribution of observed IVF outcomes specifically to sperm pre-binding to OECs. Many studies in cattle rely on frozen-thawed semen, pre-selected by Percoll gradients, which already enrich for motile and morphologically normal sperm, potentially masking the full discriminatory capacity of epithelial binding. Moreover, experimental models vary widely – from whole explants to OEC monolayers (**Table 4**) – and are usually collected from cows of unknown origin at slaughterhouse, introducing heterogeneity in cell physiology and specific ligand expression. Notably, the stage of the estrous cycle at which oviducts are collected was shown to affect sperm binding capacity *in vitro,* even after 24 h of culture (Cortat et al., 2026). Although technically easy to obtain, monolayers of OECs at confluence dedifferentiate and lose their epithelial characteristics and cilia (Sostaric et al. 2008; Schmaltz-Panneau et al., 2015), leading to unspecific sperm binding. Thus, the most physiological *in vitro* models to select sperm before IVF are oviduct explants/aggregates or spheroids. Cell-free oviduct ligands are easiest to implement routinely but this option is still limited by the lack of knowledge on *in vivo* ligands.

The above data support a central thesis: the oviduct does not passively store sperm but actively selects and conditions a functionally superior subpopulation. To date, the relative contribution of sorting through binding versus modulation of sperm physiology via cell signaling remains incompletely resolved. Sperm selection based on oviduct-inspired approaches remained largely confined to laboratory studies and have not yet resulted in commercially available selection tools for IVEP. Although beyond the scope of this review, another promising application of sperm binding assays using oviduct ligands is a better prediction of male fertility in the field. Several studies reported a greater sperm binding capacity to OECs in bulls with a higher pregnancy rate per AI compared to low-fertility bull semen (De pauw et al., 2002; Saraf et al., 2019; Donnellan et al., 2022; Silva et al., 2025).

## Conclusion

Current sperm selection methods prior to AI or IVEP or largely rely on motility- (swim-up) and morphology-based (DGC) approaches, which only partially reflect the functional competence of spermatozoa. Expanding these strategies to incorporate the complex and finely tuned behaviors exhibited by spermatozoa within the female tract represents a promising avenue for improvement. In this context, microfluidic chips designed to sort bull sperm have recently yielded encouraging results for IVF, although their throughput remains limited and restricts routine application. Further inspiration may be drawn from the female tract, which optimizes sperm survival, fertilizing ability and synchronizes gamete encounter within the oviduct. However, our understanding of key female-derived interacting factors and mechanisms governing sperm selection remains incomplete. Bridging these knowledge gaps will be essential to develop more physiologically relevant and efficient sperm sorting strategies for assisted reproduction.

## Data Availability

No research data was used.

## References

[B001] Alcântara-Neto AS, Fernandez-Rufete M, Corbin E, Tsikis G, Uzbekov R, Garanina AS, Coy P, Almiñana C, Mermillod P (2020). Oviduct fluid extracellular vesicles regulate polyspermy during porcine in vitro fertilisation. Reprod Fertil Dev.

[B002] Alghamdi AS, Lovaas BJ, Bird SL, Lamb GC, Rendahl AK, Taube PC, Foster DN (2009). Species-specific interaction of seminal plasma on sperm–neutrophil binding. Anim Reprod Sci.

[B003] Alkan H, Satilmis F, Demirel MA, Bodu M, Yesilkaya OF, Ciftci MF, Erdem H, Tekindal MA, Alkan KK (2023). Does using microfluidic sperm sorting chips in bovine IVEP affect blastocyst development?. Reprod Domest Anim.

[B004] Apichela SA, Valz-Gianinet JN, Schuster S, Jiménez-Díaz MA, Roldán-Olarte EM, Miceli DC (2010). Lectin binding patterns and carbohydrate mediation of sperm binding to llama oviductal cells in vitro. Anim Reprod Sci.

[B005] Arias ME, Andara K, Briones E, Felmer R (2017). Bovine sperm separation by Swim-up and density gradients (Percoll and BoviPure): effect on sperm quality, function and gene expression. Reprod Biol.

[B006] Ataei A, Lau AWC, Asghar W (2021). A microfluidic sperm-sorting device based on rheotaxis effect. Microfluid Nanofluidics.

[B007] Bahat A, Tur-Kaspa I, Gakamsky A, Giojalas LC, Breitbart H, Eisenbach M (2003). Thermotaxis of mammalian sperm cells: a potential navigation mechanism in the female genital tract. Nat Med.

[B008] Batista RI, Moro LN, Corbin E, Alminana C, Souza-Fabjan JM, de Figueirêdo Freitas VJ, Mermillod P (2016). Combination of oviduct fluid and heparin to improve monospermic zygotes production during porcine in vitro fertilization. Theriogenology.

[B009] Ben L, Yan C, Si-jiu Y (2013). Effect of swim-up and percoll treatment on sperm quality and in vitro embryo development in yak. J Integr Agric.

[B010] Bergqvist AS, Rodríguez-Martínez H (2006). Sulphated glycosaminoglycans (S-GAGs) and syndecans in the bovine oviduct. Anim Reprod Sci.

[B011] Bosch P, de Avila JM, Ellington JE, Wright RW (2001). Heparin and Ca2+-free medium can enhance release of bull sperm attached to oviductal epithelial cell monolayers. Theriogenology.

[B012] Bourke D.A., Lindsay F.E.F. (1988). Uterine contractions in the cow and associated movement of inseminate and other substances.

[B013] Bragança GM, Alcântara-Neto AS, Batista RITP, Brandão FZ, Freitas VJF, Mermillod P, Souza-Fabjan JMG (2021). Oviduct fluid during IVF moderately modulates polyspermy in in vitro-produced goat embryos during the non-breeding season. Theriogenology.

[B014] Bureau M, Bailey JL, Sirard MA (2000). Influence of oviductal cells and conditioned medium on porcine gametes. Zygote.

[B015] Camara Pirez M, Li S, Koelle S (2021). Assessment of sperm binding capacity in the tubal reservoir using a bovine ex vivo oviduct culture and fluorescence microscopy. Methods Protoc.

[B016] Canovas S, Romar R, Grullon LA, Aviles M, Coy P (2009). Pre-fertilization zona pellucida hardening by different cross-linkers affects IVF in pigs and cattle and improves embryo production in pigs. Reproduction.

[B017] Chinnasamy T, Kingsley JL, Inci F, Turek PJ, Rosen MP, Behr B, Tüzel E, Demirci U (2018). Guidance and self-sorting of active swimmers: 3D periodic arrays increase persistence length of human sperm selecting for the fittest. Adv Sci (Weinh).

[B018] Cortat PR, de Souza CA, Bridi A, E Silva LO, Balistrieri M, Schneberger F, Celeghini EC, da Silveira JC, Carvalho JO, Sartori R (2026). Spermatozoa binding to oviduct cells: the influence of the endocrine milieu on the effectiveness of the binding test. Theriogenology.

[B019] De Pauw IM, Van Soom A, Laevens H, Verberckmoes S, de Kruif A (2002). Sperm binding to epithelial oviduct explants in bulls with different nonreturn rates investigated with a new in vitro model. Biol Reprod.

[B020] Ded L, Hwang JY, Miki K, Shi HF, Chung JJ (2020). 3D in situ imaging of the female reproductive tract reveals molecular signatures of fertilizing spermatozoa in mice. eLife.

[B021] Denissenko P, Kantsler V, Smith DJ, Kirkman-Brown J (2012). Human spermatozoa migration in microchannels reveals boundary-following navigation. Proc Natl Acad Sci USA.

[B022] Dobrowolski W, Hafez ES (1970). Transport and distribution of spermatozoa in the reproductive tract of the cow. J Anim Sci.

[B023] Dominguez EM, Moreno-Irusta A, Guidobaldi HA, Tribulo H, Giojalas LC (2018). Improved bovine in vitro embryo production with sexed and unsexed sperm selected by chemotaxis. Theriogenology.

[B024] Donnellan EM, Lonergan P, Meade KG, Fair S (2022). An ex-vivo assessment of differential sperm transport in the female reproductive tract between high and low fertility bulls. Theriogenology.

[B025] Dutta S, Aoki K, Doungkamchan K, Tiemeyer M, Bovin N, Miller DJ (2019). Sulfated Lewis A trisaccharide on oviduct membrane glycoproteins binds bovine sperm and lengthens sperm lifespan. J Biol Chem.

[B026] Ealy AD, Wooldridge LK, McCoski SR (2019). Board Invited Review: post-transfer consequences of in vitro-produced embryos in cattle. J Anim Sci.

[B027] Eisenbach M, Giojalas LC (2006). Sperm guidance in mammals: an unpaved road to the egg. Nat Rev Mol Cell Biol.

[B028] Eisenbach M (1999). Mammalian sperm chemotaxis and its association with capacitation. Dev Genet.

[B029] Eisenbach M (2025). Sperm navigation in humans: a concerted action of multiple means. Commun Biol.

[B030] Ellington JE, Evenson DP, Wright RW, Jones AE, Schneider CS, Hiss GA, Brisbois RS (1999). Higher-quality human sperm in a sample selectively attach to oviduct (fallopian tube) epithelial cells in vitro. Fertil Steril.

[B031] Elliott RM, Lloyd RE, Fazeli A, Sostaric E, Georgiou AS, Satake N, Watson PF, Holt WV (2009). Effects of HSPA8, an evolutionarily conserved oviductal protein, on boar and bull spermatozoa. Reproduction.

[B032] El-Sherry TM, Elsayed M, Abdelhafez HK, Abdelgawad M (2014). Characterization of rheotaxis of bull sperm using microfluidics. Integr Biol (Camb).

[B033] El-Sokary MMM, Shehata SF, Mahmoud KGM (2022). Heparin and progesterone exert synergistic effects to improve the in-vitro fertilization rate of bovine sperm bound to oviduct cell aggregates from the isthmus. Vet Sci.

[B034] Fair S, Meade K, Reynaud K, Druart X, de Graaf SP (2019). The biological mechanisms regulating sperm selection by the ovine cervix. Reproduction.

[B035] Fujihara Y, Miyata H, Ikawa M (2018). Factors controlling sperm migration through the oviduct revealed by gene-modified mouse models. Exp Anim.

[B036] Fujihara Y, Noda T, Kobayashi K, Oji A, Kobayashi S, Matsumura T, Larasati T, Oura S, Kojima-Kita K, Yu Z, Matzuk MM, Ikawa M (2019). Identification of multiple male reproductive tract-specific proteins that regulate sperm migration through the oviduct in mice. Proc Natl Acad Sci USA.

[B037] García-Vázquez FA, Gadea J, Matás C, Holt WV (2016). Importance of sperm morphology during sperm transport and fertilization in mammals. Asian J Androl.

[B038] García-Vázquez FA, Hernández-Caravaca I, Matás C, Soriano-Úbeda C, Abril-Sánchez S, Izquierdo-Rico MJ (2015). Morphological study of boar sperm during their passage through the female genital tract. J Reprod Dev.

[B039] Gervasi MG, Osycka-Salut C, Sanchez T, Alonso CA, Llados C, Castellano L, Franchi AM, Villalón M, Perez-Martinez S (2016). Sperm release from the oviductal epithelium depends on Ca(2+) influx upon activation of CB1 and TRPV1 by anandamide. J Cell Biochem.

[B040] Gil MA, Ruiz M, Cuello C, Vazquez JM, Roca J, Martinez EA (2004). Influence of sperm: oocyte ratio during in vitro fertilization of in vitro matured cumulus-intact pig oocytes on fertilization parameters and embryo development. Theriogenology.

[B041] Gimeno BF, Bariani MV, Laiz-Quiroga L, Martínez-León E, Von-Meyeren M, Rey O, Mutto AÁ, Osycka-Salut CE (2021). Effects of In Vitro Interactions of Oviduct Epithelial Cells with Frozen-Thawed Stallion Spermatozoa on Their Motility, Viability and Capacitation Status. Animals (Basel).

[B042] Gualtieri R, Mollo V, Barbato V, Talevi R (2010). Ability of sulfated glycoconjugates and disulfide-reductants to release bovine epididymal sperm bound to the oviductal epithelium in vitro. Theriogenology.

[B043] Gualtieri R, Talevi R (2003). Selection of highly fertilization-competent bovine spermatozoa through adhesion to the Fallopian tube epithelium in vitro. Reproduction.

[B044] Guidobaldi HA, Hirohashi N, Cubilla M, Buffone MG, Giojalas LC (2017). An intact acrosome is required for the chemotactic response to progesterone in mouse spermatozoa. Mol Reprod Dev.

[B045] Gwathmey TM, Ignotz GG, Mueller JL, Manjunath P, Suarez SS (2006). Bovine seminal plasma proteins PDC-109, BSP-A3, and BSP-30-kDa share functional roles in storing sperm in the oviduct. Biol Reprod.

[B046] Gwathmey TM, Ignotz GG, Suarez SS (2003). PDC-109 (BSP-A1/A2) promotes bull sperm binding to oviductal epithelium in vitro and may be involved in forming the oviductal sperm reservoir. Biol Reprod.

[B047] Hansen PJ (2019). Reproductive physiology of the heat-stressed dairy cow: implications for fertility and assisted reproduction. Anim Reprod.

[B048] Hawk HW (1987). Transport and Fate of Spermatozoa After Insemination of Cattle. J Dairy Sci.

[B049] Heidarnejad A, Sadeghi M, Arasteh S, Ghiass MA (2025). A novel microfluidic device for human sperm separation based on rheotaxis. Zygote.

[B050] Heyman Y, Levasseur MC, Constantin A., Meissonnier E (1981). L’utérus de la vache: Anatomie, Physiologie, Pathologie.

[B051] Hino T, Yanagimachi R (2019). Active peristaltic movements and fluid production of the mouse oviduct: their roles in fluid and sperm transport and fertilization. Biol Reprod.

[B052] Hourcade JD, Pérez-Crespo M, Fernández-González R, Pintado B, Gutiérrez-Adán A (2010). Selection against spermatozoa with fragmented DNA after postovulatory mating depends on the type of damage. Reprod Biol Endocrinol.

[B053] Huang J, Chen H, Li N, Zhao Y (2023). Emerging microfluidic technologies for sperm sorting. Eng Regen.

[B054] Huang VW, Zhao W, Lee CL, Lee CY, Lam KK, Ko JK, Yeung WS, Ho PC, Chiu PC (2013). Cell membrane proteins from oviductal epithelial cell line protect human spermatozoa from oxidative damage. Fertil Steril.

[B055] Hughes JR, McMorrow KJ, Bovin N, Miller DJ (2023). An oviduct glycan increases sperm lifespan by diminishing the production of ubiquinone and reactive oxygen species. Biol Reprod.

[B056] Hunter RH, Fléchon B, Fléchon JE (1991). Distribution, morphology and epithelial interactions of bovine spermatozoa in the oviduct before and after ovulation: a scanning electron microscope study. Tissue Cell.

[B057] Hunter RH, Nichol R (1986). A preovulatory temperature gradient between the isthmus and ampulla of pig oviducts during the phase of sperm storage. J Reprod Fertil.

[B058] Hunter RH (1984). Pre-ovulatory arrest and peri-ovulatory redistribution of competent spermatozoa in the isthmus of the pig oviduct. J Reprod Fertil.

[B059] Huszar G, Jakab A, Sakkas D, Ozenci CC, Cayli S, Delpiano E, Ozkavukcu S (2007). Fertility testing and ICSI sperm selection by hyaluronic acid binding: clinical and genetic aspects. Reprod Biomed Online.

[B060] Ignotz GG, Cho MY, Suarez SS (2007). Annexins are candidate oviductal receptors for bovine sperm surface proteins and thus may serve to hold bovine sperm in the oviductal reservoir. Biol Reprod.

[B061] Johnson GP, English AM, Cronin S, Hoey DA, Meade KG, Fair S (2017). Genomic identification, expression profiling, and functional characterization of CatSper channels in the bovine†. Biol Reprod.

[B062] Kadirvel G, Machado SA, Korneli C, Collins E, Miller P, Bess KN, Aoki K, Tiemeyer M, Bovin N, Miller DJ (2012). Porcine sperm bind to specific 6-sialylated biantennary glycans to form the oviduct reservoir. Biol Reprod.

[B063] Karimi A, Jiang X, Abbaspourrad A (2026). Navigation and selection of spermatozoa in a radial flow microfluidic device. Lab Chip.

[B064] Katila T (2001). Sperm–uterine interactions: a review. Anim Reprod Sci.

[B065] Kawano N, Araki N, Yoshida K, Hibino T, Ohnami N, Makino M, Kanai S, Hasuwa H, Yoshida M, Miyado K, Umezawa A (2014). Seminal vesicle protein SVS2 is required for sperm survival in the uterus. Proc Natl Acad Sci USA.

[B066] Khalil AA, Petrunkina AM, Sahin E, Waberski D, Töpfer-Petersen E (2006). Enhanced binding of sperm with superior volume regulation to oviductal epithelium. J Androl.

[B067] Kikuchi K, Iisaka S, Uriu R, Shinohara H, Ezaki A, Shimada R, Fujimura S, Tani N, Araki K, Noda T, Ishiguro KI (2026). TEX30 safeguards sperm morphogenesis and quality consistency required for male fertility. Reproduction.

[B068] Kon Y, Iwata H, Shiono H, Matsubara K, Kurita A, Sakaguchi Y, Kuwayama T, Monji Y (2009). Effect of carbohydrates on the ability of bull sperm to bind to bovine oviduct epithelial cells. Reprod Domest Anim.

[B069] Kumar V, Kumaresan A, Kumar D S P, Lathika S, Nayak S, Kishor Saraf K, Nag B S P, Chhillar S, Kumar Datta T, Kumar Mohanty T (2017). Anandamide exerts a suppressive effect on sperm binding to oviduct explants through CB1 receptors in the water buffalo (Bubalus bubalis). Anim Reprod Sci.

[B070] La Spina FA, Puga Molina LC, Romarowski A, Vitale AM, Falzone TL, Krapf D, Hirohashi N, Buffone MG (2016). Mouse sperm begin to undergo acrosomal exocytosis in the upper isthmus of the oviduct. Dev Biol.

[B071] Lamy J, Corbin E, Blache MC, Garanina AS, Uzbekov R, Mermillod P, Saint-Dizier M (2017). Steroid hormones regulate sperm-oviduct interactions in the bovine. Reproduction.

[B072] Lamy J, Liere P, Pianos A, Aprahamian F, Mermillod P, Saint-Dizier M (2016). Steroid hormones in bovine oviductal fluid during the estrous cycle. Theriogenology.

[B073] Larasati T, Noda T, Fujihara Y, Shimada K, Tobita T, Yu Z, Matzuk MM, Ikawa M (2020). Tmprss12 is required for sperm motility and uterotubal junction migration in mice. Biol Reprod.

[B074] Larsson B, Larsson K (1985). Distribution of spermatozoa in the genital tract of artificially inseminated heifers. Acta Vet Scand.

[B075] Leemans B, Gadella BM, Sostaric E, Nelis H, Stout TA, Hoogewijs M, Van Soom A (2014). Oviduct binding and elevated environmental ph induce protein tyrosine phosphorylation in stallion spermatozoa. Biol Reprod.

[B076] Li J, Ning B, Cao X, Luo Y, Guo L, Wei G, Liu S, Zhang Y, Zhang A, Wu R, Li Y (2016). Separation of motile sperm for in vitro fertilization from frozen-thawed bull semen using progesterone induction on a microchip. Anim Reprod Sci.

[B077] Li S, Winuthayanon W (2017). Oviduct: roles in fertilization and early embryo development. J Endocrinol.

[B078] Lonergan P, Fair T (2014). The ART of studying early embryo development: progress and challenges in ruminant embryo culture. Theriogenology.

[B079] López-Úbeda R, García-Vázquez FA, Gadea J, Matás C (2017). Oviductal epithelial cells selected boar sperm according to their functional characteristics. Asian J Androl.

[B080] Lucio AC, Resende MV, Dernowseck-Meirelles JA, Perini AP, Oliveira LZ, Miguel MCV, Carmo AS, Tomita SY, Alves BCA, Fazano FAT, Lima VFMH (2012). Assessment of swim-up and discontinuous density gradient in sperm sex preselection for bovine embryo production. Arq Bras Med Vet Zootec.

[B081] Lyons A, Narciandi F, Donnellan E, Romero-Aguirregomezcorta J, Farrelly CO, Lonergan P, Meade KG, Fair S (2018). Recombinant β-defensin 126 promotes bull sperm binding to bovine oviductal epithelia. Reprod Fertil Dev.

[B082] Machado SA, Kadirvel G, Daigneault BW, Korneli C, Miller P, Bovin N, Miller DJ (2014). LewisX-containing glycans on the porcine oviductal epithelium contribute to formation of the sperm reservoir. Biol Reprod.

[B083] Mahé C, Lavigne R, Com E, Pineau C, Zlotkowska AM, Tsikis G, Mermillod P, Schoen J, Saint-Dizier M (2023). The sperm-interacting proteome in the bovine isthmus and ampulla during the periovulatory period. J Anim Sci Biotechnol.

[B084] Mahé C, Marco L, Laffont L, Demattei MV, Reynaud K, Com E, Lavigne R, Pineau C, Saint-Dizier M (2025). Bovine ampullary and isthmic epithelial spheroids: proteomic profile and physiological features for in vitro studies of gamete-oviduct interactions. Reprod Biol.

[B085] Mahé C, Mowinska AM, Albrecht E, Reynaud K, Lavigne R, Com E, Pineau C, Mermillod P, Saint-Dizier M, Schoen J (2025). Laser capture proteomics reveals new candidates for sperm-interacting proteins in the bovine oviduct epithelium. Reproduction.

[B086] Mahé C, Pranomphon T, Reynaud K, Laffont L, Meylheuc T, Schoen J, Mermillod P, Saint-Dizier M (2023). Sperm-fluid-cell interplays in the bovine oviduct: glycosaminoglycans modulate sperm binding to the isthmic reservoir. Sci Rep.

[B087] Malverdi N, Kazemi S, Tavakoli N, Deemeh MR, Abedinzadeh M, Vahidi S, Salehi P (2026). A special perspective on human fertilization: The role of sperm capacitation proteins and channels: a narrative review. Int J Reprod Biomed.

[B088] Marco L., Laffont L., Mahe C., Mermillod P., Bonnet-Garnier A., Saint-Dizier M. (2025). Pre-binding to isthmic epithelial spheroids enhances bull sperm ability to fertilize oocytes and obtain blastocysts in vitro.

[B089] Marey MA, Aboul Ezz M, Akthar I, Yousef MS, Imakawa K, Shimada M, Miyamoto A (2020). Sensing sperm via maternal immune system: a potential mechanism for controlling microenvironment for fertility in the cow. J Anim Sci.

[B090] Marey MA, Ma D, Yoshino H, Elesh IF, Zinnah MA, Fiorenza MF, Moriyasu S, Miyamoto A (2023). Sperm induce proinflammatory responses in the uterus and peripheral blood immune cells of artificially inseminated cows. J Reprod Dev.

[B091] Mashiko D, Tonai S, Ikawa M (2026). ADAM5 is required for sperm-zona pellucida binding and sperm oviduct migration. Biol Reprod.

[B092] Mattner PE (1968). The distribution of spermatozoa and leucocytes in the female genital tract in goats and cattle. J Reprod Fertil.

[B093] Miki K, Clapham DE (2013). Rheotaxis guides mammalian sperm. Curr Biol.

[B094] Miller DJ (2026). From reservoir to rendezvous: the journey of sperm through the oviduct. Reprod Fertil Dev.

[B095] Miller DJ (2018). Review: the epic journey of sperm through the female reproductive tract. Animal.

[B096] Moore HD, Taggart DA (1995). Sperm pairing in the opossum increases the efficiency of sperm movement in a viscous environment. Biol Reprod.

[B097] Muro Y, Hasuwa H, Isotani A, Miyata H, Yamagata K, Ikawa M, Yanagimachi R, Okabe M (2016). Behavior of mouse spermatozoa in the female reproductive tract from soon after mating to the beginning of fertilization. Biol Reprod.

[B098] Nag P, Kumaresan A, Akshaya S, Manimaran A, Rajendran D, Paul N, Sharma A, Karuthadurai T, Kaustubh S, Jeyakumar S, Ramesha K (2021). Sperm phenotypic characteristics and oviduct binding ability are altered in breeding bulls with high sperm DNA fragmentation index. Theriogenology.

[B099] Nagashima K, Usui T, Baba T (2019). Behavior of ACRBP-deficient mouse sperm in the female reproductive tract. J Reprod Dev.

[B100] Nagata MPB, Endo K, Ogata K, Yamanaka K, Egashira J, Katafuchi N, Yamanouchi T, Matsuda H, Goto Y, Sakatani M, Hojo T, Nishizono H, Yotsushima K, Takenouchi N, Hashiyada Y, Yamashita K (2018). Live births from artificial insemination of microfluidic-sorted bovine spermatozoa characterized by trajectories correlated with fertility. Proc Natl Acad Sci USA.

[B101] Noda T, Uriu R, Mashiko D, Shinohara H, Qu Y, Taira A, Matzuk RM, Tahala D, Nakano M, Araki K, Yu Z, Zhang Y, Matzuk MM, Ikawa M (2025). GALNTL5 binds GalNAc and is required for migration through the uterotubal junction and sperm-zona pellucida binding. Nat Commun.

[B102] Nosrati R, Vollmer M, Eamer L, San Gabriel MC, Zeidan K, Zini A, Sinton D (2014). Rapid selection of sperm with high DNA integrity. Lab Chip.

[B103] Oren-Benaroya R, Kipnis J, Eisenbach M (2007). Phagocytosis of human post-capacitated spermatozoa by macrophages. Hum Reprod.

[B104] Oren-Benaroya R, Orvieto R, Gakamsky A, Pinchasov M, Eisenbach M (2008). The sperm chemoattractant secreted from human cumulus cells is progesterone. Hum Reprod.

[B105] Osycka-Salut CE, Castellano L, Fornes D, Beltrame JS, Alonso CAI, Jawerbaum A, Franchi A, Díaz ES, Perez Martinez S (2017). Fibronectin from oviductal cells fluctuates during the estrous cycle and contributes to sperm-oviduct interaction in cattle. J Cell Biochem.

[B106] Parrish JJ, Krogenaes A, Susko-Parrish JL (1995). Effect of bovine sperm separation by either swim-up or Percoll method on success of in vitro fertilization and early embryonic development. Theriogenology.

[B107] Parrish JJ, Susko-Parrish JL, Handrow RR, Sims MM, First NL (1989). Capacitation of bovine spermatozoa by oviduct fluid. Biol Reprod.

[B108] Pensabene V, Agate F, Santos Miranda A, Picton HM (2026). Microfluidics for in vitro fertilization: from science to clinical validation. Hum Reprod Update.

[B109] Pérez-Cerezales S, Laguna-Barraza R, de Castro AC, Sánchez-Calabuig MJ, Cano-Oliva E, de Castro-Pita FJ, Montoro-Buils L, Pericuesta E, Fernández-González R, Gutiérrez-Adán A (2018). Sperm selection by thermotaxis improves ICSI outcome in mice. Sci Rep.

[B110] Phiphattanaphiphop C, Leksakul K, Phatthanakun R, Khamlor T (2020). A novel microfluidic chip-based sperm-sorting device constructed using design of experiment method. Sci Rep.

[B111] Qu Y, Chen Q, Guo S, Ma C, Lu Y, Shi J, Liu S, Zhou T, Noda T, Qian J, Zhang L, Zhu X, Lei X, Cao Y, Li W, Li W, Plachta N, Matzuk MM, Ikawa M, Duan E, Zhang Y, Wang H (2021). Cooperation-based sperm clusters mediate sperm oviduct entry and fertilization. Protein Cell.

[B112] Ramal-Sanchez M, Bernabo N, Tsikis G, Blache MC, Labas V, Druart X, Mermillod P, Saint-Dizier M (2020). Progesterone induces sperm release from oviductal epithelial cells by modifying sperm proteomics, lipidomics and membrane fluidity. Mol Cell Endocrinol.

[B113] Rickard JP, Pool KR, Druart X, de Graaf SP (2019). The fate of spermatozoa in the female reproductive tract: A comparative review. Theriogenology.

[B114] Rivera-Concha R, Moya C, León M, Uribe P, Schulz M, Prado A, Taubert A, Hermosilla C, Sánchez R, Zambrano F (2023). Effect of different sperm populations on neutrophils extracellular traps (NETs) formation in cattle. Res Vet Sci.

[B115] Romero-Aguirregomezcorta J, Cronin S, Donnellan E, Fair S (2019). Progesterone induces the release of bull spermatozoa from oviductal epithelial cells. Reprod Fertil Dev.

[B116] Romero-Aguirregomezcorta J, Laguna-Barraza R, Fernández-González R, Štiavnická M, Ward F, Cloherty J, McAuliffe D, Larsen PB, Grabrucker AM, Gutiérrez-Adán A, Newport D, Fair S (2021). Sperm selection by rheotaxis improves sperm quality and early embryo development. Reproduction.

[B117] Roy D, Levi K, Kiss V, Nevo R, Eisenbach M (2020). Rhodopsin and melanopsin coexist in mammalian sperm cells and activate different signaling pathways for thermotaxis. Sci Rep.

[B118] Ruiz-Díaz S, Mazzarella R, Navarrete-López P, Fernández-González R, de Frutos C, Maroto M, Cucala C, Beltrán-Breña P, Lombó M, Rizos D, Gutiérrez-Adán A (2023). Bull spermatozoa selected by thermotaxis exhibit high DNA integrity, specific head morphometry, and improve ICSI outcome. J Anim Sci Biotechnol.

[B119] Ryu H, Nam K, Lee BE, Jeong Y, Lee S, Kim J, Hyun YM, Kim JI, Park JH (2024). The sperm hook as a functional adaptation for migration and self-organized behavior. eLife.

[B120] Saint-Dizier M, Souza-Fabjan JMG, Reynaud K, Mermillod P, Almiñana C, Bauersachs S, Mahé C (2025). Oviduct epithelium interactions: roles in sperm selection and embryo quality. Anim Reprod.

[B121] Samardzija M, Karadjole M, Getz I, Makek Z, Cergolj M, Dobranic T (2006). Effects of bovine spermatozoa preparation on embryonic development in vitro. Reprod Biol Endocrinol.

[B122] Saraf KK, Singh RK, Kumaresan A, Nayak S, Chhillar S, Lathika S, Datta TK, Mohanty TK (2019). Sperm functional attributes and oviduct explant binding capacity differs between bulls with different fertility ratings in the water buffalo (Bubalus bubalis). Reprod Fertil Dev.

[B123] Schmaltz L, Prudhomme T, Tsikis G, Reynaud K, Mérour I, Mermillod P, Saint-Dizier M (2024). Sperm binding to oviduct epithelial spheroids varies among males and ejaculates but not among females in pigs. Theriogenology.

[B124] Schmaltz-Panneau B, Locatelli Y, Uzbekova S, Perreau C, Mermillod P (2015). Bovine oviduct epithelial cells dedifferentiate partly in culture, while maintaining their ability to improve early embryo development rate and quality. Reprod Domest Anim.

[B125] Silva MA, Cortat PR, Consentini CEC, Pinto SCC, Celeghini ECC, Carvalho JO, Melo LF, Wiltbank M, Sartori R (2025). Predicting sire fertility in artificial insemination of dairy cows by the ability of spermatozoa to bind to oviduct cell aggregates. J Dairy Sci.

[B126] Somfai T, Bodó S, Nagy S, Papp AB, Iváncsics J, Baranyai B, Gócza E, Kovács A (2002). Effect of swim up and Percoll treatment on viability and acrosome integrity of frozen–thawed bull spermatozoa. Reprod Domest Anim.

[B127] Sostaric E, Dieleman SJ, van de Lest CH, Colenbrander B, Vos PL, Garcia-Gil N, Gadella BM (2008). Sperm binding properties and secretory activity of the bovine oviduct immediately before and after ovulation. Mol Reprod Dev.

[B128] Soto-Heras S, Volz LJ, Bovin N, Miller DJ (2025). Porcine sperm bind to an oviduct glycan coupled to glass surfaces as a model of sperm interaction with the oviduct. Sci Rep.

[B129] Suarez SS (2008). Regulation of sperm storage and movement in the mammalian oviduct. Int J Dev Biol.

[B130] Suga T, Higaki S (1971). Studies on uterine secretions in the cow. II. Distribution of spermatozoa and seminal plasma after intra-uterine inseminations in the reproductive tract of the cow during oestrus. Bulletin of the National Institute of Animal Industry.

[B131] Tecle E, Reynoso HS, Wang R, Gagneux P (2019). The female reproductive tract contains multiple innate sialic acid-binding immunoglobulin-like lectins (Siglecs) that facilitate sperm survival. J Biol Chem.

[B132] Teijeiro J.M., Munuce M.J., Caille A.M., Zumoffen C., Marini P.E. (2017). Use of Annexin V based sperm selection in assisted reproduction. Andrology.

[B133] Tollner TL, Yudin AI, Tarantal AF, Treece CA, Overstreet JW, Cherr GN (2008). Beta-defensin 126 on the surface of macaque sperm mediates attachment of sperm to oviductal epithelia. Biol Reprod.

[B134] Tung CK, Ardon F, Fiore AG, Suarez SS, Wu M (2014). Cooperative roles of biological flow and surface topography in guiding sperm migration revealed by a microfluidic model. Lab Chip.

[B135] Vallet-Buisan M, Mecca R, Jones C, Coward K, Yeste M (2023). Contribution of semen to early embryo development: fertilization and beyond. Hum Reprod Update.

[B136] Vega-Hidalgo J, Rodriguez M, Dipaz-Berrocal D, Rivas J, Huayhua C, Mellisho E (2022). Sperm selection techniques in cattle: microfilter device versus conventional methods. Andrologia.

[B137] Viana JH (2025). 2024 Statistics of embryo production and transfer in domestic farm animals. Embryo Technology Newsletter.

[B138] Wang Z, Wei H, Wu Z, Zhang X, Sun Y, Gao L, Zhang W, Su YQ, Zhang M (2022). The oocyte cumulus complex regulates mouse sperm migration in the oviduct. Commun Biol.

[B139] Ward F, Rizos D, Corridan D, Quinn K, Boland M, Lonergan P (2001). Paternal influence on the time of first embryonic cleavage post insemination and the implications for subsequent bovine embryo development in vitro and fertility in vivo. Mol Reprod Dev.

[B140] Warr S, Pini T, de Graaf SP, Rickard JP (2023). Molecular insights to the sperm-cervix interaction and the consequences for cryopreserved sperm. Biol Reprod.

[B141] Weng L (2019). IVF-on-a-chip: recent advances in microfluidics technology for in vitro fertilization. SLAS Technol.

[B142] Wilmut I, Hunter RH (1984). Sperm transport into the oviducts of heifers mated early in estrus. Reprod Nutr Dev.

[B143] Wongtawan T, Dararatana N, Thongkittidilok C, Kornmatitsuk S, Oonkhanond B (2020). Enrichment of bovine X-sperm using microfluidic dielectrophoretic chip: a proof-of-concept study. Heliyon.

[B144] Wu JK, Chen PC, Lin YN, Wang CW, Pan LC, Tseng FG (2017). High-throughput flowing upstream sperm sorting in a retarding flow field for human semen analysis. Analyst.

[B145] Wu Z, Li B, Yu K, Zheng N, Yuan F, Miao J, Zhang M, Wang Z (2023). The mature COC promotes the ampullary NPPC required for sperm release from porcine oviduct cells. Int J Mol Sci.

[B146] Xiao W, Yu M, Yuan Y, Liu X, Chen Y (2022). Thermotaxis of mammalian sperm. Mol Hum Reprod.

[B147] Xiong W, Wang Z, Shen C (2019). An update of the regulatory factors of sperm migration from the uterus into the oviduct by genetically manipulated mice. Mol Reprod Dev.

[B148] Yaghoobi M, Abdelhady A, Favakeh A, Xie P, Cheung S, Mokhtare A, Lee YL, Nguyen AV, Palermo G, Rosenwaks Z, Cheong SH, Abbaspourrad A (2024). Faster sperm selected by rheotaxis leads to superior early embryonic development in vitro. Lab Chip.

[B149] Yamaguchi R, Muro Y, Isotani A, Tokuhiro K, Takumi K, Adham I, Ikawa M, Okabe M (2009). Disruption of ADAM3 impairs the migration of sperm into oviduct in mouse1. Biol Reprod.

[B150] Yamaguchi R, Yamagata K, Ikawa M, Moss SB, Okabe M (2006). Aberrant distribution of ADAM3 in sperm from both angiotensin-converting enzyme (Ace)- and Calmegin (Clgn)-Deficient Mice. Biol Reprod.

[B151] Yániz JL, Lopez-Gatius F, Santolaria P, Mullins KJ (2000). Study of the functional anatomy of bovine oviductal mucosa. Anat Rec.

[B152] Yazdan Parast F, Gaikwad AS, Prabhakar R, O’Bryan MK, Nosrati R (2023). The cooperative impact of flow and viscosity on sperm flagellar energetics in biomimetic environments. Cell Rep Phys Sci.

[B153] Yuan L, Ge T, Yang L, Xu W, Li G, Xu L, Zhao Y, Cheng X, Lu W, Meng S, Zhao J, Yang F, Niu C, Zheng Y (2025). Disruption of TEX38 impairs sperm morphogenesis and the migration of sperm into the oviduct. Commun Biol.

[B154] Zaferani M, Cheong SH, Abbaspourrad A (2018). Rheotaxis-based separation of sperm with progressive motility using a microfluidic corral system. Proc Natl Acad Sci USA.

[B155] Zambrano F, Carrau T, Gärtner U, Seipp A, Taubert A, Felmer R, Sanchez R, Hermosilla C (2016). Leukocytes coincubated with human sperm trigger classic neutrophil extracellular traps formation, reducing sperm motility. Fertil Steril.

